# Seismic Response of Star-Type Grid Concrete Wall Structure by Numerical Modeling

**DOI:** 10.3390/ma15238519

**Published:** 2022-11-29

**Authors:** Baizan Tang, Yuying Dong, Wen Bai, Hua-Peng Chen, Haiyang Zhuang, Wenchao Deng

**Affiliations:** 1 Key Laboratory of Earthquake Engineering and Engineering Vibration, Institute of Engineering Mechanics, China Earthquake Administration, Harbin 150086, China; 2 Key Laboratory of Earthquake Disaster Mitigation, Ministry of Emergency Management, Harbin 150086, China; 3 State Key Laboratory of Performance Monitoring and Protecting of Rail Transit Infrastructure, East China Jiaotong University, Nanchang 330013, China; 4 Institute of Geotechnical Engineering, Nanjing Tech University, Nanjing 210009, China

**Keywords:** star-type grid concrete wall, seismic response, numerical simulation, deformation

## Abstract

Cement polystyrene shell mold (CPSM) grid concrete walls have been widely applied in the construction of low and mid-rise buildings with higher load-bearing and insulation properties. A star-type grid concrete wall was constructed based on the infill wall simplified to an equivalent diagonal bracing model. To investigate the seismic responses and behavior of a star-type grid concrete wall structure, an overall time-history numerical simulation was carried out in this paper. Typical results, including acceleration, deformation, hysteresis curve and failure pattern of this novel construction system, were interpreted. Results indicate that the star-type grid concrete wall structure has satisfactory seismic performance, including energy dissipation capacity. The structure has higher lateral stiffness and can work in an elastic state under major earthquakes. Accordingly, it is more sensitive to near-fault ground motion with higher frequency components. Meanwhile, the structural inter-story drift angle is less than the limit value of lighter damage when subjected to a super-major earthquake, and the structure presents shear deformation. The openings significantly affect the failure mode, the star-type grid concrete wall with a window (a small aspect ratio less than 1.11) conforms to shear failure, and the wall with a door (aspect ratio of 2.5) conforms to bending-shear failure. The diagonal bracing can distribute the stress in the wall, especially the concrete lattice beam, and effectively resist the lateral forces via the concrete lattice column, improving the ductility and integrity of the structural system.

## 1. Introduction

**Figure 1:**
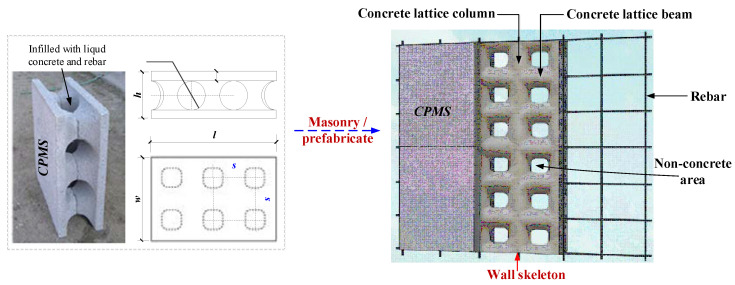
CPMS grid concrete wall.

The cement polystyrene shell mold (CPSM) grid concrete wall is a new type of wall that integrates thermal insulation, energy saving, fire resistance, lightweight, load bearing, sound insulation, environmental protection and convenient construction [1,2,3,4], consistent with the policy of reforming wall materials in China [2]. The wall is gradually used in low- and multi-story buildings. CPMS is made of cement, waste polystyrene particles, fly ash, water and modified additives. CPMS of standard specification is constructed into wall panels at staggered joints at the construction site or prefabricated in the factory. Then, steel bars are configured in horizontal and vertical core holes of these CPMS, and high fluidity self-compacting vibration-free concrete is poured to form a grid-like concrete composite wall composed of transverse limbs (‘concrete lattice beam’) and vertical limbs (‘concrete lattice column’), as shown in Figure 1. The CPMS can be used as the template of the cast-in-place grid concrete wall in the construction stage and as the thermal insulation and fireproof material of the building exterior wall in the use stage. Hence, it is in line with the 14th Five-Year Building Energy Conservation and Green Building Development Plan in China and the draft fib Model Code 2020 [5] recommendations for designing construction works with low or no carbon impact, such as improving the efficiency of material usage, structural forms and a series of common problems existing in wall insulation materials. Meanwhile, the grid concrete wall has the advantages of good integrity, fast construction speed and low project cost, and conforms to the sustainability principle of concrete structures [6,7].

In recent years, numerical modeling and model tests have been performed to investigate the seismic response of concrete wall structures. Xu et al. [8] constructed a reinforced concrete frame structure using the OpenSees software to investigate seismic fragility. By using finite element software ABAQUS, Asadi et al. [9] researched the response modification factor due to the ductility of the screen grid insulating concrete form wall. By combination with the results of pseudo-static tests, Zhao [10] conducted a non-linear finite element analysis on EPS grid frame to explain its seismic failure mechanism. Zhou et al. [11] and Lopez et al. [12] conducted monotonic and cyclic in-plane lateral loading tests on screen-grid insulated concrete rectangular and T-shaped form walls and provided an analytical model to estimate the nominal moment of the wall. Zhang et al. [13] established a finite element model to investigate the effects of strain rates on the seismic responses of reinforced concrete structures with ABAQUS platform, and the numerical results were validated by the experimental data of the shaking table test. Tang et al. [14,15] carried out shaking table tests and, in numerical studies on the seismic performance of reinforced concrete frames with latticed concrete infill walls, the simulated acceleration, displacement response, and tensive damage of the model structure have good agreement with the test records. Jiang et al. [16] performed a numerical simulation to analyze the seismic performance of superimposed reinforced concrete shear wall, the distribution of concrete damage, steel reinforcement stress distribution, and hysteretic curves in the numerical simulation were basically consistent with the results of the pseudo-static test. However, the research on grid concrete wall structure mainly focuses on its wall specimens, the loading method is limited to the monotonic and cyclic in-plane lateral loading, and investigations on seismic response of grid concrete wall structures are rarely reported.

This paper proposes a Star-type Grid Concrete Wall based on a simplified mechanical model. Accordingly, an efficient numerical method has been established with FEM software Abaqus to investigate the seismic behaviors and responses of star-type grid concrete wall structures with five-stories. Some conclusions and new findings are obtained. The results from this study can provide scientific guidance for the seismic design of grid concrete wall structures and promote the application of grid concrete walls in buildings.

## 2. Description of Star-Type Grid Concrete Wall and Its Structure

**Figure 2:**
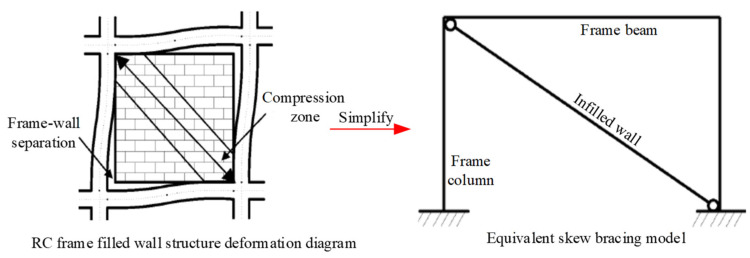
Structural mechanical model of RC frame infilled wall under horizontal load [19].

**Figure 3:**
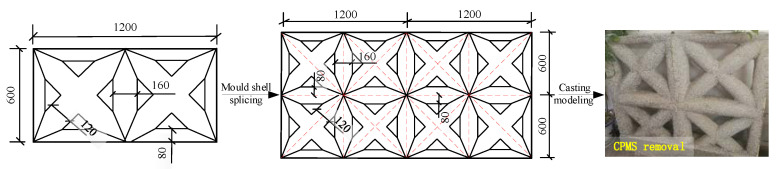
Schematic diagram of CPMS ‘star-type’ grid concrete wall.

The stress between the frame and the infilled wall interacts and transfers only in the compression area of the wall corner under a horizontal load. The infilled wall can be simplified as an equivalent skew bracing model, with only compression but no tension in the diagonal direction, hinging and cooperating with the frame plane to resist the combined lateral forces [17,18,19,20], as shown in Figure 2. Since reciprocating force is an essential characteristic of the structure under seismic loads, the infill wall is simplified to an equivalent diagonal bracing model. Combined with the shaking table test of traditional grid concrete wall structure carried out in the previous research work [15], the CPMS ‘star-type’ grid concrete wall is constructed, as shown in Figure 3. The dimension of the star-type CPMS is 1200 mm × 600 mm × 250 mm. The nominal diameter of the concrete lattice beam, concrete lattice column and diagonal bracing is 160 mm, 160 mm, and 120 mm, respectively, and the spacing of the concrete lattice beams or columns is 600 mm.

**Figure 4:**
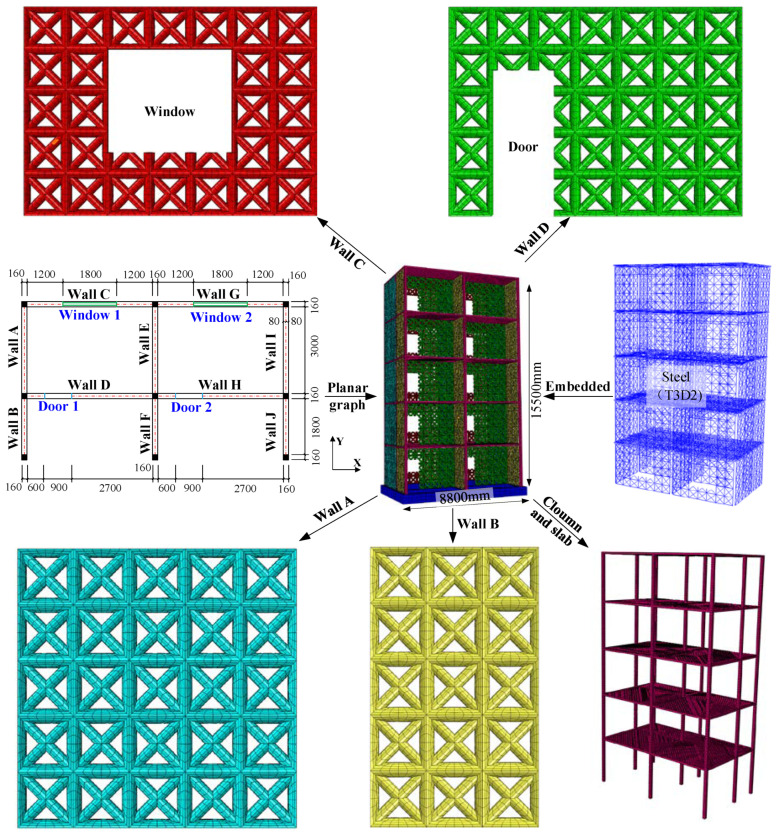
Finite element meshes of the star-type grid concrete wall structure (unit: mm).

The model structure is a five-story two-span star-type grid concrete wall structure, and the definitions of x-, y-direction, and plane dimensions of the model structure are given in Figure 4. The cross-section of the column is 160 mm × 160 mm, and the thickness of each slab is 100 mm. The height of each layer is 3.1 m, and the structural height is 15.5 m. The standard value of concrete cube compressive strength is 30.0 MPa for the grid wall structure. Meanwhile, the steel rebar of HRB400 has a design value of 360 MPa strength and a nominal diameter of 14 mm embedded in the grid wall and column. The steel rebar of HPB300 has a design value of 270 MPa strength and a nominal diameter of 8 mm embedded in the slab. To investigate the effect of aspect ratio on the failure mode of the grid concrete walls, a window and a door are set in wall C and wall D, respectively. The aspect ratio on both sides of the opening is 2.5 for wall C, 5 and 1.1 for wall D. Wall A and wall B have no holes, with aspect ratios of 1.0 and 1.67, respectively. Considering that both sides of the opening are weak parts, the arrangement of vertical reinforcement is enhanced.

## 3. Numerical Simulation

### 3.1. Numerical Model

To investigate the seismic performance of the star-type grid concrete wall structure, a 3-D finite element (FE) model is established by Abaqus software version 6.14, as shown in Figure 4. In this numerical model, eight-node hexahedron linear reduction integral hourglass control elements are selected to discretize the star-type grid concrete wall structure with the Arbitrary Lagrangian-Eulerian (ALE) adaptive meshing method. A two-node linear 3-D truss element is used to model the steel rebar embedded into the concrete. To study the influence of finite element meshing on the simulation results, the coarse and fine meshes are adapted in numerical modeling. The New-mark direct integral method is used to solve the finite element dynamics problem. To ensure the calculation accuracy, the integral time step Δt is generally determined by the first mode of structural vibration and sampling frequency of a seismic wave. In this paper, the integral time step Δt is 5 × 10^−3^ s. The damping ratio of the structure is taken as 5%, according to the Chinese code for the design of steel-concrete mixed structure of tall buildings (CECS230-2008) [21]. Rayleigh damping assumes that the damping matrix is constructed by the mass matrix and the stiffness matrix in proportion, Namely,

(1) $$C=\eta \left[M\right]+\delta \left[K\right]$$

where *C*, *M,* and *K* are the damping matrix, mass matrix and stiffness matrix, respectively, the parameter *η* denotes a coefficient proportional to the mass and the parameter *δ* denotes a coefficient proportional to the stiffness. Accordingly, the parameters *η* and *δ* can be calculated as:

(2) $$\left\{\begin{array}{l} \eta \\ \delta \end{array}\right\}=\frac{2\xi }{\omega_{i}+\omega_{j}}\left\{\begin{array}{l} \omega_{i}\omega_{j}\\ 1 \end{array}\right\}$$

where the parameter *ξ* denotes the damping ratio of the fundamental mode and *ω_i_* and *ω_j_* represent the *i*-th and *j*-th order natural vibration frequency of the structure.

### 3.2. Constitutive Model for Concrete and Steel

**Figure 5:**
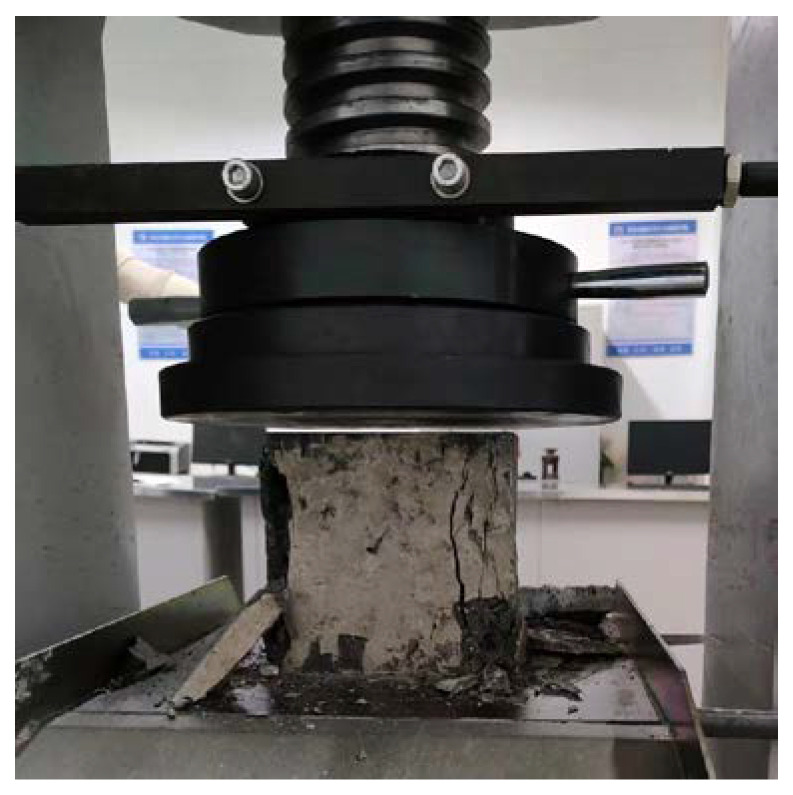
Compression test of concrete specimen.

**Figure 6:**
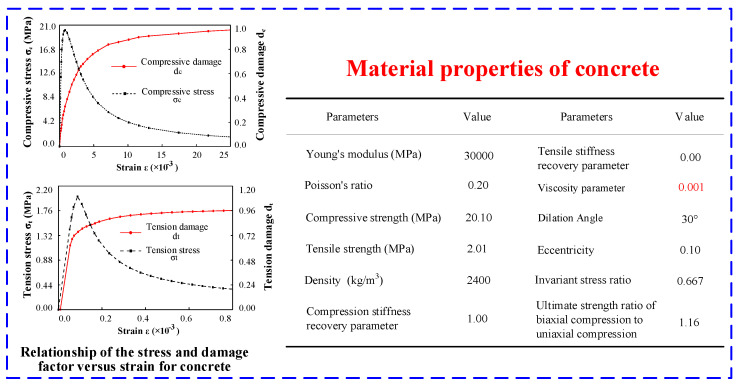
Material properties of the concrete structure.

The plastic damage constitutive model presented by Jeeho and Fenves [22] is selected to describe the nonlinear properties of concrete. It is believed that tensile cracking and compression crushing are the two main failure mechanisms of concrete and its material properties based on a laboratory test (see Figure 5); the standard value of concrete axial compressive strength is approximately 20.1 MPa for the ‘star-type’ grid concrete wall structure, and the fracture toughness and fracture energy of the concrete is 0.773 $\mathrm{MPa}\sqrt{m}$ and 151.1 N/m. Accordingly, The mix ratio of the concrete is designed as cement:flyash:water:sand:aggregates = 1.00:0.25:0.56:3.21:2.86 and the *W/C* ratio is 0.56. The properties parameters of concrete, the concrete stress-strain and damage-strain curves are shown in Figure 6. The value of the viscosity parameter is in the range of 0.001 to 0.005 [16,23]. Meanwhile, the numerical results show that, if the value of the viscosity parameter is larger, the model structure tends to become rigid. If the value is smaller, the analysis calculation is more difficult to converge. If the numerical calculation has no difficulty in convergence, the value of the viscosity parameter can be 0. In this paper, the viscosity parameter is set as 0.001.

**Figure 7:**
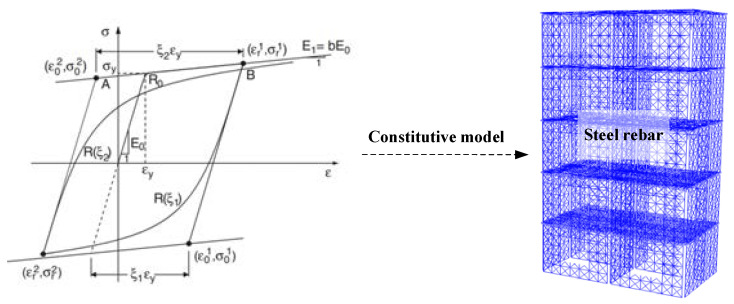
The modified Menegotto-Pinto constitutive model [24].

Considering the isotropic-hardening effects, the modified Menegotto-Pinto constitutive model [24,25] is adopted to simulate the dynamic behavior of the steel bar in this study, as shown in Figure 7. The stress–strain relationship represents the transition curve from a straight asymptote with a slope of *E*_0_ (initial elastic modulus) to another asymptote with a slope of *E*_1_. Where b = *E*_1_/*E*_0_ denotes the strain strengthening coefficient, *R*_0_ is the curvature parameter affecting the shape of the transition curve from elastic to plastic-hardening. This model can accurately and efficiently reflect the Bauschinger effect under cyclic loads.

### 3.3. Input of Ground Motions

**Figure 8:**
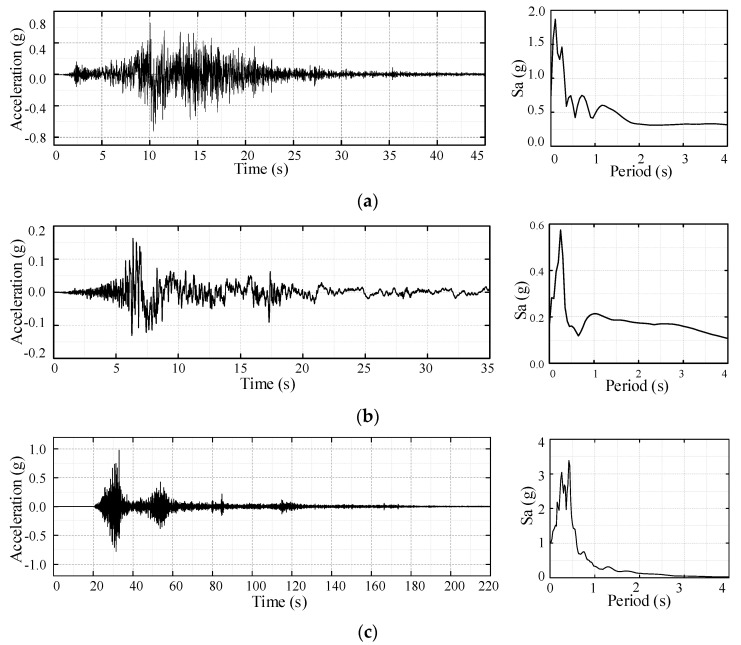
Acceleration time history and spectral acceleration of the input motions. (**a**) Lander ground motion [27], (**b**) Imperial Valley ground motion [28], (**c**) Wolong ground motion [29].

The appropriate selection of ground motion requires spectrum analysis of the structure to obtain the structural dynamic characteristics, such as damping, and natural vibration frequency. In this study, the ground motion is applied only in the X-direction and the fundamental frequency of the structure is 7.85 Hz based on the Lanczos method in the X-direction. Accordingly, three different ground motions, e.g., the Lander wave, Imperial Valley wave and Wolong wave, are chosen as the input motions in the numerical simulation. The Lander wave is a near-field strong seismic wave with a fling-step effect recorded at the Lucerne station in 1992 Landers earthquake Mw7.28, with a surface rupture length of 85 km, surficial offsets of 6 m, peak acceleration of 711.38 gal and duration of 40 s [26,27]. The Imperial Valley wave is a near-field seismic wave with directional effect recorded at the Brawley Airport station in 1979 Imperial Valley earthquake Mw76.5, with a rupture distance of 8 km, peak acceleration of 159.52 gal, and duration of 37.9 s [28]. The Wolong record was recorded at No. 051 WCW station in 2008 Wenchuan Earthquake Ms8.0, with a rupture distance of 23 km, peak acceleration of 957.7 gal, and duration of 180.0 s [29]. To simulate the seismic performance of star grid concrete wall structure under different seismic intensities, the PGA of original ground motion is adjusted to 0.1 g, 0.22 g, 0.4 g, and 0.62 g, respectively, according to the Chinese code for design of concrete structures GB50010-2010. Figure 8 displays the acceleration time history and response spectrum of the motions.

## 4. Seismic Response of the Star-Type Grid Concrete Wall Structure

### 4.1. Acceleration Response

**Figure 9:**
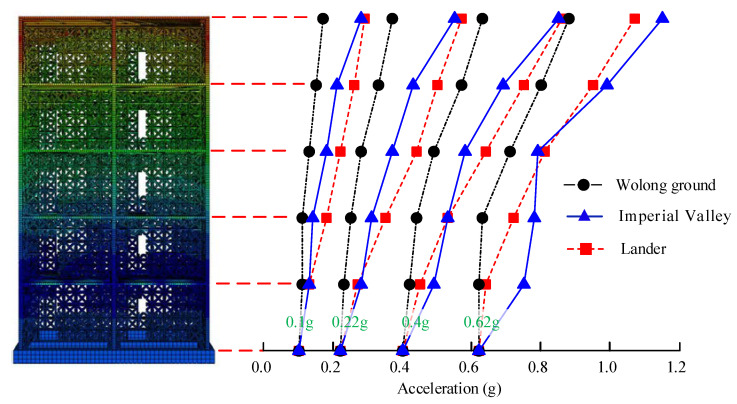
Acceleration response of the structure.

**Figure 10:**
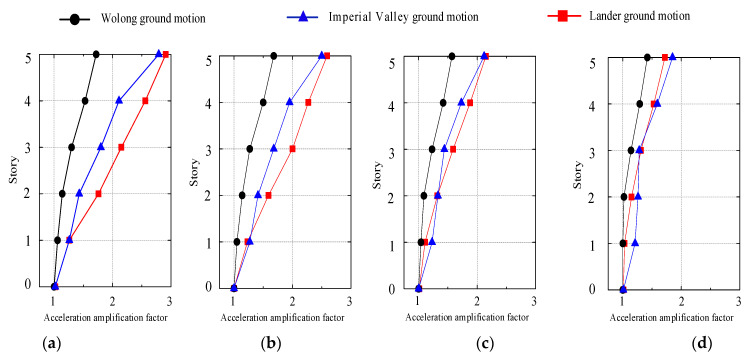
Acceleration amplification factor of the structure under different seismic intensities. (**a**) PGA = 0.1 g, (**b**) PGA = 0.22 g, (**c**) PGA = 0.4 g, (**d**) PGA = 0.62 g.

Figure 9 depicts the peak acceleration of the star-type grid concrete wall structure, and Figure 10 shows the acceleration amplification factor *K* (ratio of the maximum absolute acceleration of each story to the maximum acceleration of the input motion) of the structure. As the peak ground acceleration (PGA) of the input motion increases from 0.10 to 0.62 g, the peak acceleration of each story increases, and the maximum acceleration response is on the top story, whereas *K* decreases gradually. In the sequence of minor earthquakes (e.g., PGA = 0.10 g), the acceleration response increases linearly with the structural height, and *K* reaches 3.0 on the top story. When subjected to the rare earthquakes with a seismic intensity of degree 7 (PGA = 0.22 g), the distribution rule of the acceleration response is similar to that under minor earthquakes, and *K* is slightly reduced to 2.6, decreasing by about only 10.3%, indicating that the lateral force resisting members of the star-type grid concrete wall structure seldom damaged. As the seismic intensity increases to degree 8 (PGA = 0.40 g), *K* continues to decrease on the same story, especially in Lander ground motion, reflecting that the structural lateral stiffness decreases under strong ground motions. When subjected to a rare earthquake with a seismic intensity of degree 9 (PGA = 0.62 g), *K* is reduced to 1.79 on the top story, decreasing by about 38.3%, indicating that some lateral force-resisting members of the star-type grid concrete wall structure have already been damaged. The results coincided well with the theory and shaking table test performed by Li et al. (2019) [30] for an RC shear wall structure and Qiao et al. (2021) [31] for an insulated sandwich concrete wall building structure. 

The acceleration response of the star-type grid concrete wall structure is different under the Wolong, Lander, and Imperial Valley record inputs with the same level. Taking the top story as an example, with the increase of the input peak acceleration, the amplification factor decreases from 1.72 to 1.42 induced by the Wolong record, 2.91 to 1.72 induced by the Lander record and 2.79 to 1.85 induced by the Imperial Valley record, accordingly. The acceleration response of each story induced by the near-fault ground motion (e.g., Lander or Imperial Valley record) input is greater than those in the Wolong record inputs. The results indicate that seismic responses of the star-type grid concrete wall structure are more sensitive to the near-fault ground motion with higher frequency components. When subjected to moderate earthquakes (e.g., PGA = 0.22 g), the acceleration responses induced by the Lander record input are greater than those in the Imperial Valley record inputs. When subjected to super-major earthquakes (e.g., PGA = 0.62 g), the acceleration responses induced by the Lander record input are gradually smaller than those in the Imperial Valley record inputs, particularly at the lower story. Meanwhile, the acceleration responses below three stories are close to one another under the Imperial Valley record inputs. This is because the acceleration response is significantly related to the frequency component of the input motion and structural damage, the structural stiffness decreases due to the damage of the lateral force-resisting members of the star-type grid concrete wall structure, especially at the lower story, subjected to major earthquakes, and the Imperial Valley ground motion has lower frequency components compared to the lander ground motion. Hence, the acceleration response of the star-type grid concrete wall structure behaves according to different rules under different seismic intensities, and the inflection point is seismic intensity degree 9 (PGA = 0.62 g).

### 4.2. Displacement Response

**Figure 11:**
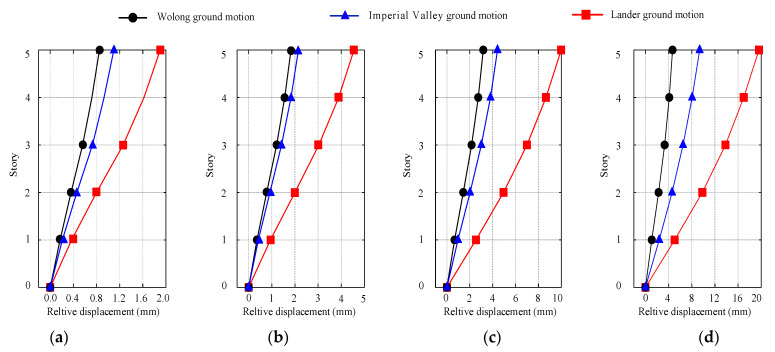
The maximum relative displacement response of stories under ground motions input. (**a**) PGA = 0.1 g, (**b**) PGA = 0.22 g, (**c**) PGA = 0.4 g, (**d**) PGA = 0.62 g.

Figure 11 depicts the maximum displacement of each story relative to the base subjected to input motions. The displacement of the structure increases with structural height, and the star-type grid concrete wall structure exhibits shear deformation. The effect of the near-fault waves, especially the lander ground motion with the fling-step effect, is more prominent than that of the other wave. The top story’s maximum displacement and displacement angle increase with the seismic intensity increases, and the maximum displacement of the top story is approximately 20 mm under a rare earthquake with a seismic intensity of degree 9, and its corresponding displacement angle is 1/775. The results meet the requirements of the Chinese codes (JGJ3-2010; GB50011-2010) [32,33].This indicates that the star-type grid concrete wall structure has greater lateral stiffness and relatively good integrity under major earthquakes without collapsing. 

**Figure 12:**
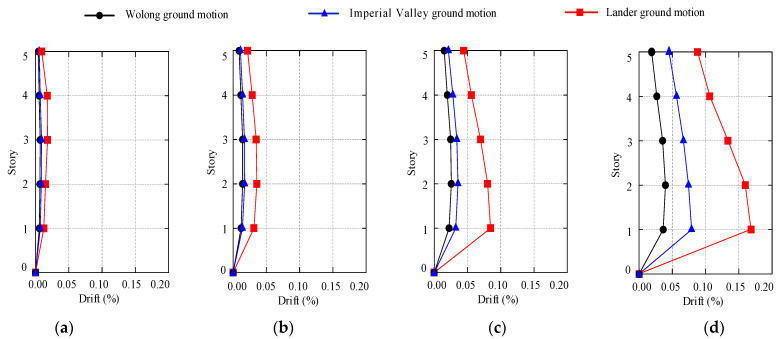
The maximum inter-story drift ratio of stories under different ground motions. (**a**) PGA = 0.1 g, (**b**) PGA = 0.22 g, (**c**) PGA = 0.4 g, (**d**) PGA = 0.62 g.

Figure 12 displays each story’s maximum inter-story drift angle *θ*_max_ under different ground motions input. Table 1 lists the seismic performance level of shear wall structures, according to the Chinese code (GB50010-2011) [33] and recent studies (Jiang et al. 2021; Qiao et al. 2021) [16,31]. As seen in Figure 11 and Table 1, the inter-story drift angle increases initially and later decreases with the structural height, and the maximum inter-story drift angle presents at the first story. When subjected to rare earthquakes with a seismic intensity of degree 8 (PGA = 0.4 g), the inter-story drift angle is less than 1%, corresponding to Level I. This indicates that the star-type grid concrete wall structure is basically intact. The maximum inter-story drift angle is 1.6% under rare earthquakes with a seismic intensity of degree 9 (PGA = 0.62 g), which is less than that of 1.8% corresponding to Level II. This suggests that the structural components are slightly damaged, particularly in the first story. The simulated result is consistent with the deformation response of concrete wall structure presented in the shaking table test (He et al. 2018; Qiao et al. 2021) [31,34]: The inter-story drift angle of the first floor is always the largest, and the stiffness of the first floor needs to be improved. It can be concluded that the degradation of the structural seismic performance level is relatively slow and the star-type grid concrete wall structure has satisfactory stiffness and ductility.

### 4.3. Hysteresis Curve

**Figure 13:**
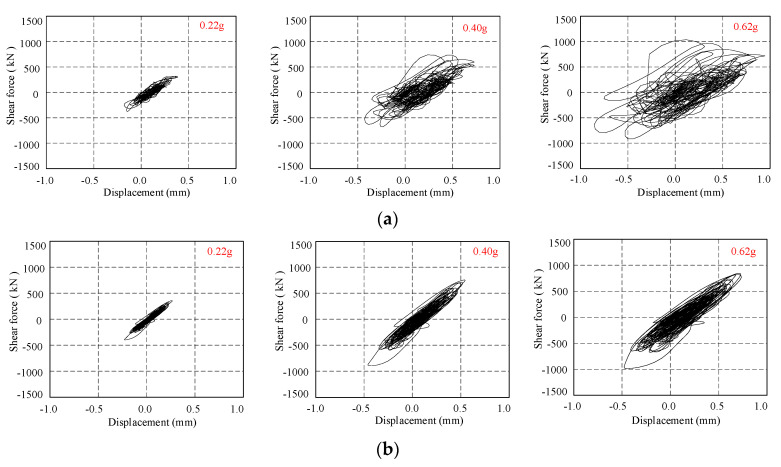
Hysteresis curve of the star-type grid concrete wall structure. (**a**) Lander ground motions, (**b**) Wolong ground motions.

As the shear response can be calculated from the mass distribution of the story and the corresponding acceleration response, the hysteresis curve of the star-type grid concrete wall structure can be obtained by the displacement response and shear response, shown in Figure 13. When subjected to a rare earthquake with a seismic intensity of degree 9 (PGA = 0.62 g), the peak shear force of the structure is 1036.5 kN and 846.3 kN for Lander ground motion and Wolong ground motion, respectively. It is noted that the hysteresis curve of the structure changes with the seismic intensity, while stiffness is not apparent; the star-type grid concrete wall structure is in the elastic range without much hysteretic energy dissipation under rare earthquakes with a seismic intensity of degree 8 (PGA = 0.40 g), and even for degree 9 (PGA = 0.62 g) under Wolong ground motion. However, the hysteresis curve becomes irregular when subjected to the Lander ground motion with a seismic intensity of degree 9 (PGA = 0.62 g), indicating that some structural components suffer damage and the star-type grid concrete wall structure is sensitive to the near-fault ground motion.

### 4.4. Failure Pattern of the Star-Type Grid Concrete Wall

**Figure 14:**
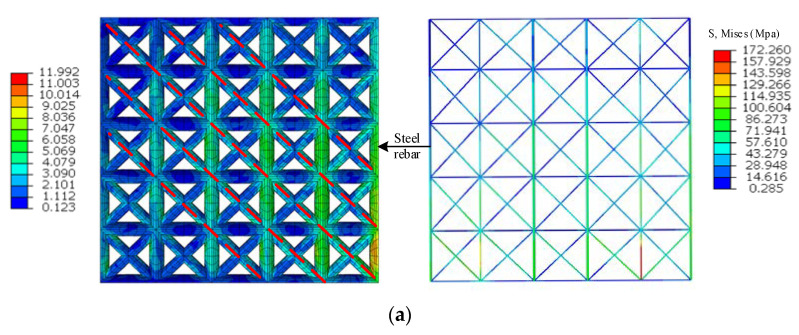

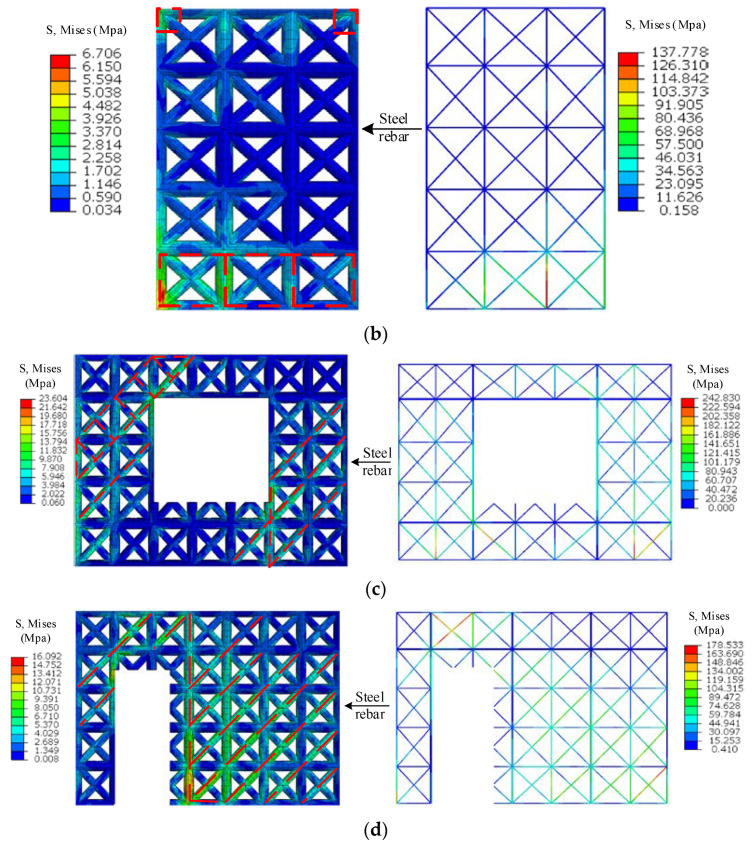
Stress contours of steel rebar under Lander ground motion with PGA = 0.62 g. (**a**) WallA, (**b**) WallB, (**c**) Wall C, (**d**) Wall D.

Considering the first story suffers maximum inter-story drift angle, Figure 14 shows the stress contours of star-type grid concrete walls in the first story under the Lander ground motion. For wall A, with an aspect ratio of 1:1, the stress is concentrated on the diagonal bracing and concrete lattice column, particularly along the diagonal structure line from the wall corner to the opposite one, conforming to the shear failure. In contrast, the stress is more minor in the concrete lattice beam. For wall B, with an aspect ratio of 5:3, the stress is primarily presented at the bottom and top of the wall, especially at the corners of the wall, while the stress is minimal in the middle of the wall, conforming to the bending failure. Compared with wall A, the stress of wall B, with a higher aspect ratio, is smaller. 

Due to the architectural requirements, the openings are used for windows or doors. To investigate the effects of openings, a rectangular window (1500 mm × 1800 mm) in Wall C and a door (900 mm × 2100 mm) in Wall C were investigated. It is noted that the stress is around the openings, including the components of diagonal bracing and concrete lattice column. The stress value is negatively correlated with the distance to the openings, reaching the maximum at the corner of the opening. For wall C, the stress of the wall on both sides of the opening (aspect ratio of 5:2) is mainly distributed in the diagonal bracing and ends of the concrete lattice column, conforming to the bending-shear failure. For wall D, the stress of the wall on the left side of the opening (aspect ratio of 1.11:1) is mainly distributed in each diagonal bracing and partial concrete lattice column, conforming to the shear failure. Meanwhile, the stress in wall C is more prominent than that in wall D, which may be related to the opening location and area in a given wall panel, further affecting the structural resistance. The stress characteristic of star-type grid concrete walls with long-narrow door openings is close to that of strong wall weak beam structures, the wall limbs on both sides of window openings avoiding short column damage. The results indicate that the internal stress generated by horizontal force is mainly endured by diagonal bracing and the concrete lattice column under ground motion input, and the diagonal bracing in the grid concrete wall can play an effective role in resisting the lateral forces.

The stress distribution of the steel rebar embedded in the star-type grid concrete wall is consistent with that of the wall, as well as the rule of the stress amplitude, verifying the correctness of the steel rebar model. Similar results were presented in experimental and numerical studies performed by Jiang et al. (2021) [16] and Wang et al. (2021) [35]. Meanwhile, it can be seen that the steel rebar in the diagonal bracing distributes the stress in the wall, especially the concrete lattice beam, and bears force, together with the steel rebars in the concrete lattice column. The steel rebars work cooperatively with concrete walls, improving the integrity of the structural system.

## 5. Conclusions

Based on the infill wall simplified to an equivalent diagonal bracing model, a star-type grid concrete wall is constructed in this paper. An advanced numerical model was crafted to model the seismic responses of star-type grid concrete wall structures. The acceleration, deformation, hysteresis curve and failure pattern of the structure were analyzed by using the developed FEM model. The conclusions and recommendations are summarized as follows:

(1) The star-type grid concrete wall structure has satisfactory seismic performance, especially in rare earthquakes with a seismic intensity of degree 9, which can be equivalent to that of a shear wall structure. The maximum displacement of the top story is approximately 20 mm and its corresponding displacement angle is 1/775 under rare earthquakes with a seismic intensity of degree 9, meeting the requirements of the Chinese codes (JGJ3-2010; GB50011-2010).
(2) The acceleration response of the star-type grid concrete wall structure behaves differently under strong seismic intensity; the inflection point is seismic intensity degree 9 (PGA = 0.62 g), and the decreasing acceleration amplification factor *K* is about 38.3% compared with the original value.
(3) The seismic response of each story induced by the near-fault ground motion, particularly under lander ground motion, is more significant than that by Wolong ground motion. The results indicate that seismic responses of the star-type grid concrete wall structure are more sensitive to near-fault ground motion with higher frequency components.
(4) The inter-story drift angle increases initially and later decreases with structural height; the maximum inter-story drift angle is present in the first story. The maximum inter-story drift angle is 1.6% under rare earthquakes, which is less than the limit value of lighter damage (1.8%), as specified in the Chinese code (GB50010-2011) and recent studies. It suggests that the star-type grid concrete wall structure has sufficient stiffness and ductility and the structural components in the first story need to be enhanced, such as increasing reinforcement ratio.
(5) The diagonal bracing and its steel rebar distribute the stress in the wall, especially the concrete lattice beam. The star-type grid concrete wall with long-narrow door openings presents a bending-shear failure, which can be equivalent to a strong wall weak beam structures; the wall limbs on both sides of window openings conform to a shear failure and can avoid short column damage. The diagonal bracing in the grid concrete wall can play an effective role in resisting lateral forces, and improving the integrity of the structural system.

To further investigate the seismic performance of star-type grid concrete wall structures, parametric study (e.g., material properties), shaking table tests and comparative analyses of different structures (e.g., reinforced concrete frame structure, reinforced concrete shear wall structure) are in progress, and the results will be reported in the future.

## Figures and Tables

**Table 1:** Seismic performance level of shear wall structures.

Seismic Performance	Limit Inter-Story Drift Angle	Damage Degree
Level Ⅰ	*θ*_max_ < 1/1000	Basically intact
Level Ⅱ	1/1000 *≤ θ*_max_ < 1.8/1000	Slight damage
Leve l Ⅲ	1.8/1000 *≤ θ*_max_ < 3.5/1000	Medium damage
Level Ⅳ	3.5/1000 *≤θ*_max_ < 8.3/1000	Severe damage
Level Ⅴ	*θ*_max_ ≥ 8.3/1000	Collapse

## Data Availability

Data is contained within the article.
